# A Novel Case of Cytomegalovirus Pneumonia in an Acquired Thrombotic Thrombocytopenic Purpura Patient Treated With Rituximab

**DOI:** 10.7759/cureus.14182

**Published:** 2021-03-30

**Authors:** Emad Kandah, Raghunandan Konda, Atefeh Kalantary, Adan Madadha, Arvind Kunadi

**Affiliations:** 1 Internal Medicine, McLaren Health Care, Flint/MSU, Flint, USA; 2 Diagnostic Medical Laboratories, Cell Therapy Center, University of Jordan, Amman, JOR; 3 Internal Medicine and Nephrology, McLaren Health Care, Flint/MSU, Flint, USA

**Keywords:** thrombotic thrombocytopenic purpura, ttp, cytomegalovirus, cmv, rituximab

## Abstract

Thrombotic thrombocytopenic purpura (TTP) is thrombotic microangiopathy that is universally fatal if not promptly recognized and treated. Standard treatment includes plasma exchange (PLEX) therapy and immunosuppression. We present a case of an 80 years old African American male with a past medical history significant for essential hypertension, chronic obstructive pulmonary disease, and a recent TTP diagnosis for which he was treated with PLEX, glucocorticoids, and rituximab. The patient presented with complaints of shortness of breath of four days duration. He was hypoxemic on presentation; other vital signs were within normal limits. The basic metabolic panel and complete blood count were unremarkable. A computed tomography (CT) of the chest with contrast showed right lower lobe segmental and subsegmental pulmonary emboli. He was initiated on intravenous heparin therapy. During hospitalization, he had progressive clinical deterioration with progressive hypoxemia. A repeat CT scan demonstrated bilateral pulmonary infiltrates. The patient underwent bronchoscopy due to concerns of opportunistic infections in view of his recent immunosuppressive treatment. Bronchoalveolar lavage revealed cytomegalovirus (CMV), and the patient was initiated on ganciclovir. CMV pneumonia has been reported after rituximab therapy in patients with lymphomas and lymphoproliferative disorders. To our knowledge, this is the first case of CMV pneumonia after rituximab therapy in a patient with TTP.

## Introduction

Thrombotic thrombocytopenic purpura (TTP) is a rare thrombotic microangiopathic disorder characterized by microangiopathic hemolytic anemia, severe thrombocytopenia, and ischemic end-organ injury due to platelet-rich thrombi. TTP results from a severe deficiency of von Willebrand factor (VWF)-cleaving metalloprotease, ADAMTS13 (a disintegrin and metalloproteinase with a thrombospondin type 1 motif, member 13). ADAMTS13 deficiency is generally acquired due to anti-ADAMTS13 autoantibodies [1].

Plasma exchange (PLEX) is the mainstay of treatment. Further therapies include glucocorticoids, rituximab, and caplacizumab. Rituximab has been shown to reduce the risk of exacerbation and relapse and may hasten response to therapy [2].

## Case presentation

**Figure 1:**
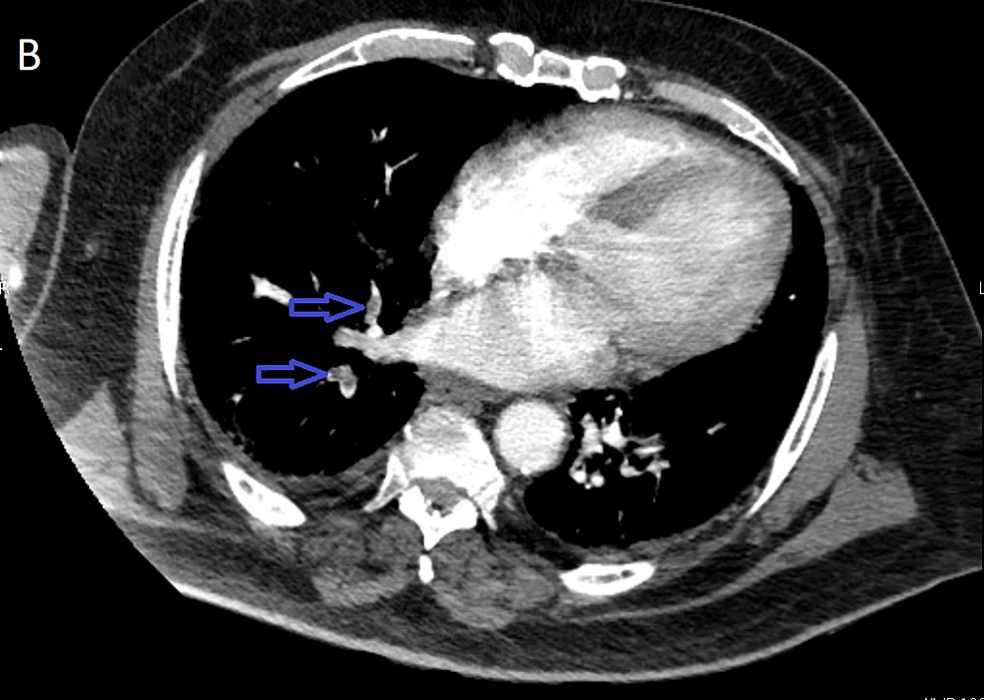
CT chest with contrast demonstrating filling defects in right lower lobe subsegmental (blue arrows) arteries suggestive of pulmonary embolism.

Our patient was an 80-year-old African American male with a past medical history significant for essential hypertension, cerebrovascular accident (CVA), chronic obstructive pulmonary disease, and recently diagnosed thrombotic thrombocytopenic purpura (TTP). He presented to the hospital with a chief complaint of shortness of breath (SOB) of 4 days duration. Upon presentation, the patient had a blood pressure of 177/83 mmHg, heart rate of 75 beats per minute, respiratory rate of 22 breaths per minute, and oxygen saturation of 96% breathing ambient air. Arterial blood gas showed a pH of 7.46 (7.35-7.45), partial pressure of carbon dioxide (PCO2) of 30.2 mmHg (35-45 mmHg), partial pressure of oxygen (PO2) of 87.7 mmHg (80-100 mmHg), and bicarbonate (HCO3) of 21.1 mmol/L (22-26 mmol/L). CT scan of the chest with contrast revealed filling defects in right lower lobe segmental and subsegmental pulmonary artery branches, consistent with acute pulmonary embolism (PE) (Figure 1). The patient was initiated on intravenous (IV) heparin therapy and was admitted to the intensive care unit for close monitoring.

The patient had a recent hospitalization for stroke-like symptoms (slurred speech, facial droop, and confusion). Stroke workup including CT head without contrast, CT perfusion of the brain, and CT angiography of the head and neck, was unremarkable at that time. Initial blood work during that admission was significant for a hemoglobin of 7.8 g/dL (13.5-17.7 g/dL), and a platelet count of 16 X 10*3/uL (140-440 X 10*3/uL). Given the anemia, thrombocytopenia, and neurologic manifestations, TTP was suspected. Further laboratory evaluation revealed a fibrinogen level of 299 mg/dL (200-400 mg/dL), haptoglobin of <8 mg/dL (13.2-198 mg/dL), and a lactate dehydrogenase (LDH) level of 654 U/L (100-225 U/L). The direct Coombs test was negative. Peripheral blood smear showed normocytic, hypochromic anemia with mild anisopoikilocytosis and 10-15 schistocytes per high-power field. Platelet count was decreased with no clumping noted. ADAMTS13 activity was < 3% (reference range 68-163%), and ADAMTS13 inhibitor level was 7.8 Bethesda equivalent units (BEU) (reference range < 0.4 BEU). The patient was started on steroid treatment as well as plasma exchange therapy (PLEX) with good response (platelet count increased to 279, with normalization of serum LDH). Response to treatment was not maintained after discontinuation of PLEX therapy, platelet count declined with a nadir of 54. PLEX therapy was restarted and the patient was initiated on rituximab. The hepatitis panel was negative prior to rituximab initiation. The patient received a total of two doses of rituximab, one week apart, in the hospital. He was discharged with a platelet count of 134, with plans to administer two additional doses in the outpatient setting.

**Figure 2:**
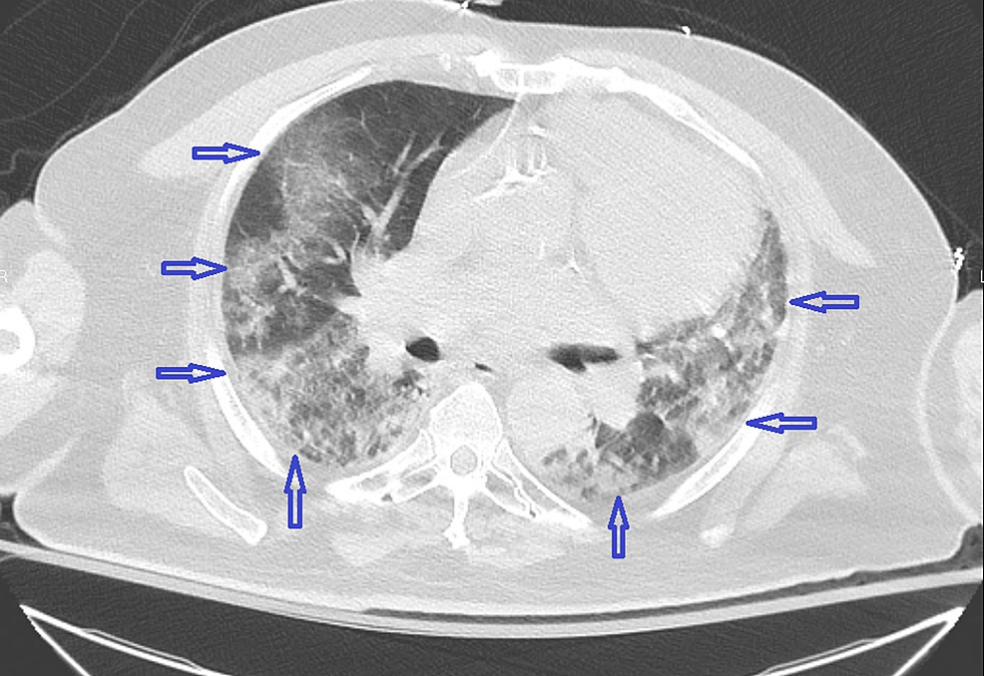
CT chest without contrast (cross-sectional view) demonstrating diffuse bilateral pulmonary opacities (blue arrows).

**Figure 3:**
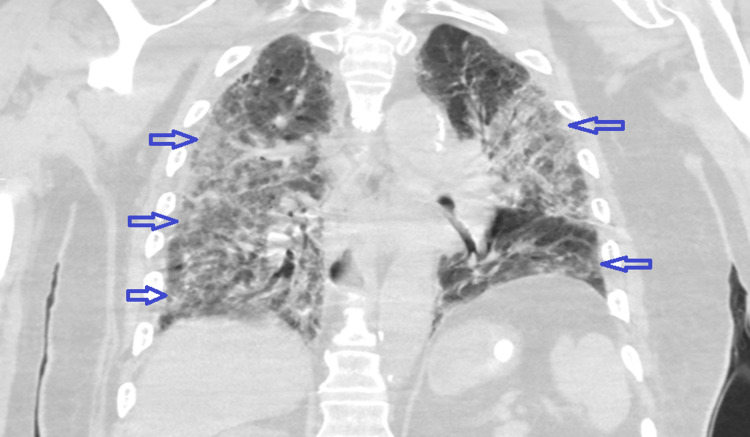
CT chest without contrast (coronal view) demonstrating diffuse bilateral pulmonary opacities (blue arrows).

In the current admission, the patient had a worsening respiratory status that necessitated escalation of oxygen support to nasal cannula, and then to high flow. Sputum culture grew *Enterobacter aerogenes*, and the patient was treated with a seven-day course of IV piperacillin/tazobactam. Due to the progressive clinical deterioration, the patient was evaluated with a repeat CT scan of the chest without contrast which showed diffuse bilateral pulmonary opacities (Figures 2, 3). Given the recent rituximab treatment and the CT scan findings, the patient was treated empirically for Pneumocystis pneumonia with trimethoprim/sulfamethoxazole that was later changed to atovaquone due to worsening renal function. The patient had a poor response to treatment with a continuing respiratory decline that necessitated endotracheal intubation and mechanical ventilation. A bronchoscopy was performed and a bronchoalveolar lavage (BAL) sample was obtained for further testing. BAL revealed herpes simplex virus type 1 (HSV1) and cytomegalovirus (CMV). The patient was initiated on ganciclovir. The patient failed multiple spontaneous breathing trials afterward, and the family decided to proceed with comfort measures. Shortly after that, the patient expired. Table 1 includes additional microbiological testing that was performed during this hospital admission, the results, and the test reference ranges.

**Table 1:** Additional microbiological testing. BAL: bronchoalveolar lavage

Test	Specimen source	Result	Reference range
Anti-nuclear antibody	Blood	Negative	Negative
Aspergillus Flavus antibody	Blood	Negative	Negative
Aspergillus Niger antibody	Blood	Negative	Negative
Aspergillus Fumigatus antibody	Blood	Negative	Negative
Aspergillus Antigen	BAL	Negative	Negative
Blastomyces Antibody	Blood	Negative	Negative
Cytomegalovirus IgM Antibody	Blood	Non-reactive	Non-reactive
Acid Fast Bacilli	BAL	Negative	Negative
Blood Culture	Blood	Negative	Negative
Legionella Culture	Sputum	Negative	Negative
Cytomegalovirus DNA, Qualitative	Blood	Detected	Not Detected
Cytomegalovirus DNA, Quantitative	Blood	682	< 126 IU/mL
Coccidioides Antibody	Blood	Not Detected	Not Detected
Histoplasma Antibody	Blood	Not Detected	Not Detected
Hepatitis A IgM antibody	Blood	Non-reactive	Non-reactive
Hepatitis B Core IgM Antibody	Blood	Non-reactive	Non-reactive
Hepatitis B Surface Antigen	Blood	Non-reactive	Non-reactive
Hepatitis B Surface Antibody	Blood	Non-reactive	Non-reactive
Hepatitis C Antibody	Blood	Non-reactive	Non-reactive
Herpes Simplex Virus type 1	Blood	Detected	Not Detected
Herpes Simplex Virus type 2	Blood	Not Detected	Not Detected
HIV 1 Antibody	Blood	Non-reactive	Non-reactive
HIV 1 P24 antigen	Blood	Non-reactive	Non-reactive
HIV 2 Antibody	Blood	Non-reactive	Non-reactive

## Discussion

Thrombotic thrombocytopenic purpura (TTP) is a rare hematologic disorder characterized by disseminated platelet-rich microthrombi with resultant microangiopathic hemolytic anemia, severe thrombocytopenia, and organ damage. The hallmark pathophysiology is represented by a deficiency of von Willebrand factor (VWF)-cleaving metalloprotease, ADAMTS13 [1, 3]. TTP is further classified based on the mechanism of ADAMTS13 deficiency into congenital and acquired TTP. Congenital TTP (inherited, also known as Upshaw-Schulman syndrome) caused by a mutation in the ADAMTS13 gene with a resultant severe deficiency (<10%) in ADAMTS13. Acquired TTP caused by anti-ADATMS13 autoantibodies [1, 4]. These antibodies can be either inhibitory, causing neutralization of ADAMTS13 proteolytic activity, or non-inhibitory that binds to ADAMTS13, accelerating its clearance from the plasma. Patients with African ancestry and female patients are more prone to develop these autoantibodies [1].

The classic pentad of acquired TTP - fever, microangiopathic hemolytic anemia, thrombocytopenia, neurological symptoms, and renal insufficiency - is present in less than 10% of cases. TTP almost uniformly presents with severe thrombocytopenia (<30 x 10^9/L) and microangiopathic hemolytic anemia characterized by schistocytes on peripheral blood smear [3]. Other hemolytic parameters are also present, including elevated indirect bilirubin, elevated serum LDH, and low haptoglobin concentration [1]. Clinical presentation is diverse and is related to organ ischemia or infarction. Almost 60% of patients have neurologic manifestations ranging from headache and confusion to stroke, seizure, or coma at presentation. Mesenteric ischemia presenting with abdominal pain, nausea, and diarrhea occur in almost 35% of patients. Renal involvement consists of hematuria and proteinuria. Though unusual, acute renal failure requiring renal replacement therapy has been reported. Myocardial ischemia is present in almost 25% of patients with acute TTP and is characterized by an abnormal electrocardiogram, or more commonly, the elevation of cardiac troponin [1, 3, 5].

TTP is a clinical emergency and once suspected, treatment should be initiated promptly to significantly reduce its morbidity and mortality. Treatment should not be delayed while awaiting confirmatory ADAMTS13 testing. Plasma exchange (PLEX) therapy remains the mainstay treatment of TTP. The postulated mechanism of plasma exchange is that it replaces the deficient ADMATS13 while removing the circulating autoantibodies. Glucocorticoids are generally used concomitantly with plasma exchange at the initiation of therapy as immunosuppressive therapy provides a more durable response than plasma exchange alone [1, 3, 5]. Our patient was initially treated with PLEX therapy and glucocorticoids.

Additional immunosuppressive agents (e.g., rituximab, caplacizumab) are usually required in patients who fail to achieve clinical response, defined as a platelet count more than 150 x 10^9/L along with normalizing LDH concentration; with frontline therapy (PLEX and glucocorticoids). These agents are also used in patients experiencing an exacerbation of the disease defined as disease recurrence within 30 days of treatment cessation after achieving clinical response, in patients with relapse defined as disease recurrence 30 days or more of treatment cessation after achieving clinical response, and in patients with refractory disease defined as persistent thrombocytopenia and persistently elevated LDH despite concurrent PLEX and steroid therapies [1, 3, 6]. Our patient had an exacerbation of TTP and was initiated on rituximab.

Rituximab is a chimeric monoclonal antibody directed against CD20 antigen present on B lymphocytes. Rituximab is widely used in the treatment of autoimmune diseases, including TTP. Its effectiveness in TTP treatment stems from B cell depletion resulting in suppression of anti-ADAMTS14 autoantibodies production [1, 7]. Rituximab is generally well tolerated with mild reactions not necessitating cessation of treatment. However, rare and life-threatening complications have been reported with rituximab therapy, such as renal toxicity, cardiovascular events, progressive multifocal leukoencephalopathy, and viral infections [8, 9]. A literature review of viral infections after rituximab therapy conducted by Aksoy et al. showed that hepatitis B virus (HBV) infection was the most common infection observed in 39% of the cases. Cytomegalovirus infection was observed in 23% of the cases [10].

Cytomegalovirus (CMV) usually is a latent infection and gets reactivated during an immunocompromised state, such as immunosuppressive medications, hypogammaglobulinemia, organ transplantation, and acute graft reaction [11-13]. Severe CMV infections associated with rituximab therapy have been reported in post-organ-transplant patients and patients with lymphomas and lymphoproliferative disorders [14].

## Conclusions

Rituximab is being increasingly prescribed by oncologists, and hepatitis B virus screening is already being performed prior to commencing treatment. Although the exact incidence of CMV infection associated with rituximab treatment has not been reported, physicians should be aware of this complication.

CMV infection in post organ transplant patients and patients with lymphomas and lymphoproliferative disorders treated with rituximab is well described in the literature. To our best knowledge, this is the first case of CMV infection in a patient with TTP treated with rituximab. This case also highlights the risk of severe and potentially fatal opportunistic infections after rituximab treatment.
